# Plasticity of signaling and mate choice in a trilling species of the *Mecopoda* complex (Orthoptera: Tettigoniidae)

**DOI:** 10.1007/s00265-017-2381-6

**Published:** 2017-10-24

**Authors:** I. Krobath, H. Römer, M. Hartbauer

**Affiliations:** Institute of Zoology, University of Graz, Universitätsplatz 2, A-8010 Graz, Austria

**Keywords:** Katydid, Female choice, Signal plasticity, Inter-male competition, Receiver preference, Complex calling song

## Abstract

**Abstract:**

Males of a trilling species in the *Mecopoda* complex produce conspicuous calling songs that consist of two motifs: an amplitude-modulated motif with alternating loud and soft segments (AM-motif) and a continuous, high-intensity trill. The function of these song motifs for female attraction and competition between males was investigated. We tested the hypothesis that males modify their signaling behavior depending on the social environment (presence/absence of females or rival males) when they compete for mates. Therefore, we analyzed acoustic signaling of males in three different situations: (1) solo singing, (2) acoustic interaction with another male, and (3) singing in the presence of a female. In addition, the preference of females for these song motifs and further song parameters was studied in two-choice experiments. As expected, females showed a preference for conspicuous and loud song elements, but nevertheless, males increased the proportion of the AM-motif in the presence of a female. In acoustic interactions, males reduced bout duration significantly compared to both other situations. However, song bouts in this situation still overlapped more than expected by chance, which indicates intentionally simultaneous singing. A multivariate statistical analysis revealed that the proportion of the AM-motif and the duration of loud segments within the AM-motif allow a reliable prediction of whether males sing in isolation, compete with another male, or sing in the presence of a female. These results indicate that the AM-motif plays a dominant role especially in close-range courtship and that males are challenged in finding a balance between attracting females and saving energy during repeated acoustic interactions.

**Significance statement:**

Males of acoustic insects often produce conspicuous calling songs that have a dual function in male-male competition and mate attraction. High signal amplitudes and signal rates are associated with high energetic costs for signal production. We would therefore predict that males adjust their signaling behavior according to their perception of the social context. Here we studied signal production and mate choice in a katydid, where males switch between loud and soft song segments in a dynamic way. Additionally, we examined the attractiveness of different song elements in female choice tests. Our results show how males of this katydid deal with the conflict of remaining attractive for females and competing with a costly signal with rivals.

## Introduction

Males of many acoustic insect species produce conspicuous calling songs to attract females from a distance and, at the same time, compete with rival males. These calling songs often exhibit high amplitudes and signal rates, which are song parameters associated with high costs. Costs for the sender arise as a result of energy investment during sound production (Prestwich 1994) and/or the risk of unintentionally attracting eavesdropping predators or parasitoids (Zuk and Kolluru 1998). Greenfield (1994a) proposed that song parameters such as high amplitude, song duration, and other exaggerated signal features are manifest expressions of competition between males, the development of which is the outcome of co-evolutionary processes between senders and receivers (see also Kokko et al. 2003 and Lailvaux and Irschick 2006).

One important factor that probably influences characteristics of signal displays is the social context in which signal production occurs. Except for duetting species, in which one sex responds to the other sex, acoustic signaling for males is rather ‘speculative’ (Zimmermann et al. 1989), since calling males cannot predict the effectiveness of their signaling until the female arrives. Males often sing in choruses with other males within earshot of each other. In this situation, they attempt to increase the attractiveness of their call relative to that of their rivals by increasing their signaling effort (Dadour 1989; Latimer 1981; Morris et al. 2002). In the presence of rivals, for example, stickleback males invest more energy in red coloration (Sin-Yeon and Velando 2014) and weakly electric fish increase pulse amplitude and phase duration of their electric signals (e.g., Gavassa et al. 2013). Further examples for an increased energy investment in signal production of males are the field cricket *Teleogryllus commodus*, which exhibits a higher calling effort which in turn reduces their life span (Callander et al. 2013) and the calling songs and investment in spermatophores of the bushcricket *Ephippiger diurnus*, which also depend on the acoustic environment and male’s age (Rebar and Greenfield 2017). Increasing signaling effort is costly for the sender due to the inefficiency of the conversion of muscle power into acoustic power (Prestwich 1994; Gerhardt and Huber 2002). Signals produced in a chorus are often more complex and include different signal components. For example in *Hyla microcephala*, females prefer multi-note over single-note calls, which stimulates the males to increase both the call rate and number of call notes in a chorus situation (Schwartz 1986). Such competitive signaling is associated with higher energetic demands (Wells and Taigen 1989). A similar preference for complex calls is exhibited by the tungara frog (*Physalaemus pustulosus),* where competing males add chucks to whine calls in order to exploit a sensory bias in the receivers, increasing the attractiveness of their calls (Ryan and Rand 1981; Ryan and Keddy-Hector 1992, but see Ron 2008). In *P. pustulosus,* the presence of females immediately affects the males’ calling song production, driving increased signal complexity (Akre and Ryan 2011).

After an arrival of or encounter with a female, however, males often begin emitting a different, less conspicuous song. The courtship song of crickets is structurally distinct from the calling song and varies in its sound pressure level (SPL), carrier frequency, and temporal pattern (Otte and Alexander 1983; Balakrishnan and Pollack 1996; Brown 1999). Male bladder grasshoppers decrease the SPL of their calling songs from about 100 dB to about 70 dB after receiving the soft reply of a female. This reduces competition with rival males that have alternative mating tactics (Alexander and van Staaden 1989). In both cases, switching from a long-distance to a short-distance signal may facilitate private communication between the sexes and therefore indicates a distinctive function of both signal types (i.e., preparing the female to be mounted during copulation). In other species with different types of songs, however, the function of such a switch is less clear (for a review on “soft signaling” see Reichard and Anderson 2015).

**Fig. 1:**
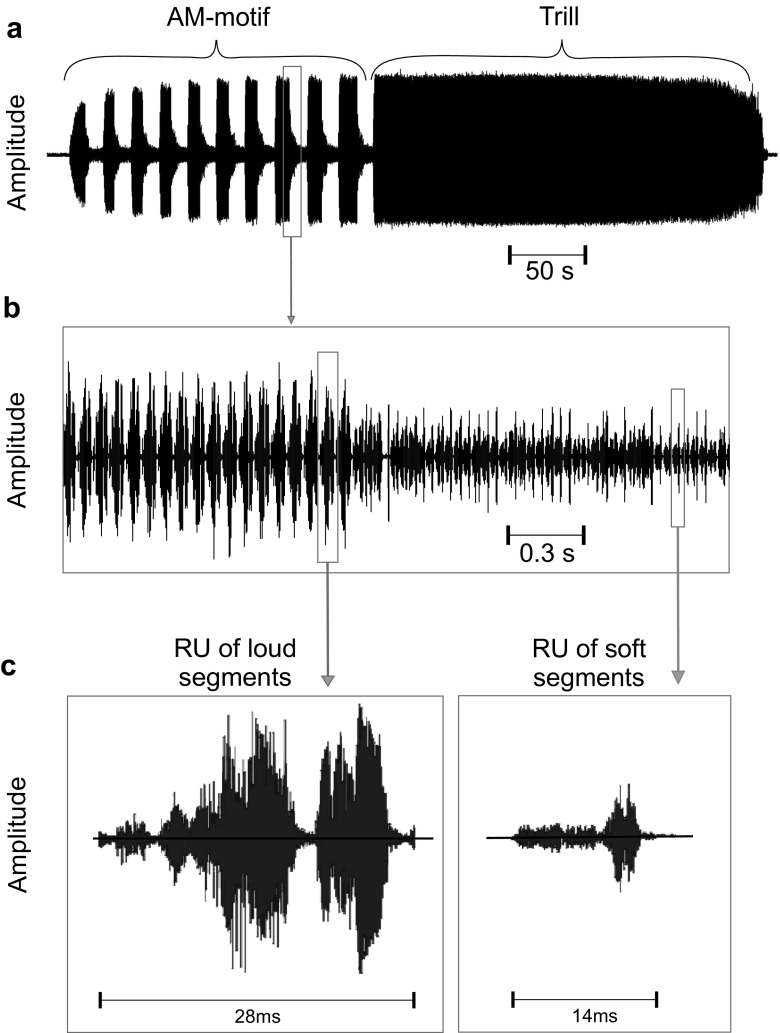
Temporal pattern of a calling song of a trilling *Mecopoda* species. **a** The song bout of a solo singing male usually consists of an amplitude-modulated motif and a trill. **b** Loud segments are made up of high amplitude syllables (left), whereas syllables of the soft segment have lower amplitudes (right). **c** Temporal pattern of repeatable units (RU) of the loud segment (left) and the soft segment (right)

In the current study, we hypothesized that males of a trilling katydid species of the *Mecopoda* complex adjust their calling behavior to the current social situation, i.e., the presence of females or competing males. These males produce complex calling songs that have alternating loud and soft song segments (AM-motif) as well as trills with a consistent, high amplitude (105 dB SPL at a distance of 15 cm, Fig. 1). The high amplitude of these calls indicates their function in long-distance mate attraction (Krobath 2013). Field recordings made in the native habitat (tropical rainforests in Malaysia) revealed that neighboring males often sing at the same time within earshot of each other. If masking by intense trills is critical in male-male competition, we would expect that males in a social context with other males increase the proportion of trills. If, however, available energy for calling is critical, males may save energy during song interactions and adjust the proportion of costly (loud) and less costly (soft) song segments in the AM-motif of the song. To test these hypotheses, we compared the calling songs produced by males singing in isolation with those of the same males when they were free to interact acoustically with a competitor. Because the two song motifs could potentially be directed toward a different audience (i.e., either female or rival male(s)), we also studied the calling songs produced by males in the presence of females and measured the amount of time spent in singing the AM-motif and trill. Similar to other species, sexual selection by female choice may act as a driving force for the evolution of complex calling songs in this species. Therefore, we studied the attractiveness of the two song motifs, duty cycles of loud segments, syllable durations, and the period durations in AM-motifs in two-choice experiments with females.

## Materials and methods

### Insects

Behavioral experiments were performed using a trilling species of the katydid *Mecopoda* sp. The taxonomy of the genus *Mecopoda* is still unresolved. Several morphologically similar sibling species have been identified that have distinctly different calling song patterns (Nityananda and Balakrishnan 2006). Insects used in this study stemmed from a laboratory breed at the Institute of Zoology in Graz, which was originally established from individuals collected from a tropical rainforest in Malaysia in 2010 and 2011. Korsunovskaya (2008) described this species as “*Mecopoda* sp. 4.” One male and female voucher specimen are deposited at the National History Museum in Vienna (NOaS-11/2013). Experiments were conducted at the Institute of Zoology, University of Graz. Insects were reared in a crowded colony in a breeding room in which the ambient temperature was 27 °C and the relative humidity 70%. Insects were fed ad libitum on fish food, oat flakes, fresh lettuce, apple pieces, and provided with water. All developmental stages were exposed to a constant light-dark cycle of 12:12 h. Males usually start singing shortly after the onset of the dark phase, during which all behavioral experiments were conducted.

### Sound recordings

Songs of males were recorded under three different conditions: when they were (1) in isolation, (2) free to interact acoustically with another male, or (3) in the presence of a female. In the first two situations, males were individually caged in wire mesh tubes that each had a cross-sectional diameter of 7.5 cm and a length of 25 cm; in the third situation, both female and male individuals were positioned on different leaves of the same plant. For the main statistics, we only considered song bouts lasting less than 1000 s, for the following reasons: median values for song bout duration were at 400 s (*Q*1 = 400 s, *Q*3 = 600 s) for interactions and 500 s (*Q*1 = 400 s, *Q*3 = 600 s) for solo singing males. Song bouts lasting longer than 1000 s (up to 7000 s) occurred with the probability of about 10%. These longer bouts were qualitatively different in that they consisted only of a continuous loud trill.

1. Solo song recordings were made in a temperature-controlled incubator (64 × 55 × 62 cm) at 27 °C. The inner surface of the incubator was covered with acoustic foam in order to minimize echoic effects and attenuate acoustic disturbances from outside. Sound recordings were taken for 4 h, starting with the onset of the dark phase, using a tie-pin microphone (Vivanco Inc., model: EM216) that was positioned 15 cm away from the singing male. Sound signals were A/D converted using a PC-internal soundcard that operated at a sampling rate of 44 kHz.
2. Recordings of song interactions were obtained from the same ten males as in (1). Each male was recorded in two to three acoustic interactions with different males (in total 17 male combinations). Interactions were recorded in a dark anechoic room (280 × 220 × 200 cm); the inner walls and ceiling were covered with sound-absorbent material. Caged males were positioned at opposite corners of this room with an inter-male distance of 2.2 m and elevation of 35 cm to the ground. The sounds produced by both males were recorded with two microphones (Voltcraft Inc., model: 33-2050) that were positioned 15 cm away from each male. The relative insensitivity of these microphones resulted in minimal crosstalk between the recordings. The microphone signals were digitized using an A/D-converter (Power1401 mk 2, Cambridge Electronic Design Limited, Cambridge, UK) that operated at a sampling rate of 20 kHz (10 kHz each channel). Ambient temperature of 27.5 ± 1 °C was maintained (thermostatic heater; Delonghi, Italy); the relative humidity was maintained between 50 and 70% using a humidifier (Bionaire CM1-l, France).
3. The songs of males singing in the presence of a female were recorded in the same dark anechoic room as in (2). An infrared-sensitive video camera (GKB CB-38075) and infrared spotlights (EuroTECH LED, 850 nm) allowed the tracking of the positions of male and female. The video was live broadcast on a screen outside the chamber, so that the approaches could be observed continuously during the experiment. To provide males with information about the presence of a nearby female, a male was placed together with a female on the same leaf of a single plant (*Strelitzia reginae*) until they came in close (antennal) contact. Then male and female were separated on different positions on the plant by placing them on different leafs, and the songs produced by males were recorded with a microphone (Voltcraft Inc., model: 33-2050) that was positioned 15 cm away from the plant. The microphone signals were digitized using a PowerLab 4/26 (AD Instruments Inc., Germany) that operated at a sampling rate of 40 kHz. Video and sound recordings were stopped when the female approached the male and started to make antennal contact, or in the case that one of the individuals left the plant. These experiments were conducted with a sample of males that was different from that in the other two situations. Thus, statistical analysis had to be adapted to this condition (see below).

### Female choice experiments

Preferences of females for various song models were studied in two-choice arena trials. The phonotaxis arena was the anechoic room described in (2) in which acoustic interactions of males were recorded. Females were released opposite two loud speakers (LEAF Tweeter Technics EAS-10TH400A, Kadoma, Japan), which were separated by 155 cm and elevated 9 cm above the ground. The arena temperature was maintained at 27 °C and the relative humidity at 70%. Trials were restricted to the dark cycle and performed in complete darkness. Females were positioned at the release point of the arena in a small wire-mesh cage and were allowed to adapt to the experimental conditions in the absence of any acoustic stimulation for about 5 min. The plug closing the cage was then removed, and acoustic stimuli were broadcast via two loudspeakers. Walking paths of females were observed and recorded from above using an infrared-setup as described in (3). The analogue output from the video camera was connected to a frame grabber (Pixelsmart Inc., USA) that stored frames at intervals of 5 s. A phonotactic approach to a speaker was regarded to be positive when females entered a virtual semicircle (radius of 30 cm) that surrounded each speaker. Choice experiments were terminated when the trials exceeded a maximum of 15 min (corresponding to 180 frames), when females stayed inside the cage, or when they immediately climbed up the wall of the arena after leaving the cage. When females were tested several times within the same dark period, a pause of at least 1 h was introduced between subsequent trials. Phonotactic tracks were reconstructed frame by frame using a motion tracking software (MTrackJ, version 1.44p), run as an ImageJ plugin (http://imagej.nih.gov/ij). The image sequence of each trial was manually analyzed by marking the position of the female in a frame by frame manner. The coordinates that coded female positions were stored in a spreadsheet and allowed the reconstruction of phonotactic walking paths.

Due to a limited number of phonotactically responsive females in some experiments, each individual was tested four times for each stimulus combination. The broadcast song models were alternated between loudspeakers to avoid a side bias.

### Playback stimuli used in two-choice experiments

Typical song bouts of *Mecopoda* sp. consist of two different motifs that differ in their amplitude modulation (Fig. 1). While the first part of a song bout is usually an amplitude modulated part (AM-motif), in which loud and soft segments alternate, the second part is mostly a trill that is produced at a consistently high amplitude. Loud segments in the AM-motif and the constant trill exhibit the same RMS amplitude and syllable structure, which is twice as long as those in soft segments. They differ only in the total duration, whereby a trill was defined as a loud segment that lasted longer than 15 s. One signal period in the AM-motif comprises one loud and one soft segment; therefore, the period duration and the duty cycle depend on the duration of loud and soft segments.

Playback stimuli were created based on calling songs that were originally recorded from males singing in isolation. Competing stimuli that were used in each trial were created by manipulating the respective parameters of a calling song of the same male. Sound recordings were made at a distance of 15 cm from singing males using a 1/2″ microphone (type 40AC, serial no. 80264; G.R.A.S. Sound & Vibration, Holte, Denmark) attached to a preamplifier (type 26AM/ type 26AC; G.R.A.S Sound & Vibration, Holte, Denmark). Its output was amplified in a Power Module (type 12AK; G.R.A.S Sound & Vibration, Holte, Denmark). A/D conversion was achieved using a fire wire sound card (type FA-101, Edirol, Roland Corporation, Tokyo, Japan) that operated at a sampling rate of 96 kHz. The same sound card which was used for A/D conversion was also used to broadcast song models through a pair of leaf tweeters. The signal amplitude was adjusted with a programmable attenuator (PA5, Tucker-Davis technologies, Florida, USA) that was connected to a signal amplifier with a linear frequency response up to 100 kHz (C272, NAD Electronics International, Ontario, Canada). For the purpose of calibration, short segments of sound signals were broadcast through the leaf tweeters in continuous loop mode. Using a sound level meter (CEL 414, Casella, Bedford, UK; 1/2″ microphone: type 2540, Larson Davis, Depew, NY, USA) that operated in the fast reading mode, signal amplitudes of loud song parts were calibrated to a SPL of 75 dB and soft parts to 62 dB SPL at the release point of females. To create different stimuli for female choice experiments, song models were manipulated using the sound editing software “Cool Edit Pro.” Manipulations of duty cycles and period durations were achieved by modifying these signal components in an original recording of an AM-motif of approximately 1-min duration.

**Fig. 2:**
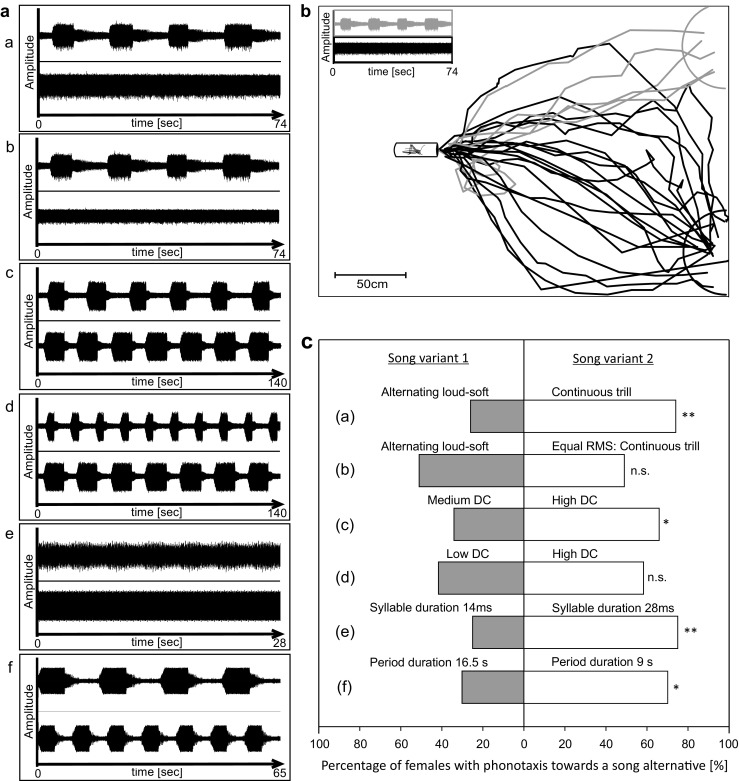
Acoustic stimulus parameter combinations and results obtained in two-choice arena trials. **a** Stimulus combinations: (a) AM-motif vs. trill, (b) AM-motif vs. trill with equal RMS, (c) medium vs. high duty cycle, (d) low duty cycle vs. high duty cycle, (e) short vs. long syllable duration, (f) long vs. short period (duration of loud and soft segment). **b** Phonotactic walking paths of females given the choice between the trill motif (lower right corner) and the AM-motif (upper corner). Semicircles indicate a distance of 30 cm from the speaker. Phonotaxis toward the trill and AM-motif of song are shown in black and gray, respectively. AM-motif and trill were broadcast in the upper and lower corners, respectively. **c** Results of two choice experiments shown as the proportion of females choosing either song variant. *indicates *p* < 0.05; **indicates *p* < 0.01

In total, seven different combinations of song models were broadcast to females in two-choice experiments (see Fig. 2). (a) Females were given a choice between the trill and the AM-motif (with a medium duty cycle of 39%), to find out whether females prefer the energetically more costly song motif over the less costly one. (b) Same as in (a) after equalizing the RMS amplitude of both stimuli, which was achieved by reducing the RMS amplitude of the trill. This experiment aims to reveal whether or not stimulus amplitude is able to overrule a general preference for a song motif. Since the duration of loud segments of AM-motifs varied between males (from 3.1 to 9.3 s), we tested the preference of females in a choice between two AM-motifs differing in the duty cycle of loud segments. We predict that females prefer songs consisting of longer, energetically demanding segments: (c) a “medium duty cycle song” of 39%, (the average duty cycle of acoustically interacting males), in a choice with a “high duty cycle song” (duty cycle of 56%). (d) The same high duty cycle song vs. a “low duty cycle song” of 27%. Due to the fact that loud and soft segments in the AM-motif also differ in their syllable structure and syllable duration (Fig. 1b, c), we offered females a choice between two different continuous trills; (e) a syllable pattern typical for soft song segments of the AM-motif vs. a pattern typical for the trill motif. Since trills are more costly to produce, we predict a preference for the syllable pattern typical for this motif. Furthermore, we tested female preferences for different signal periods, whereby one period is defined as the time encompassing one loud and one soft segment, because it is possible that songs have the same duty cycle of loud segments, but differ in the signal period. Thus, females were given a choice (f) between an AM-song model with a signal period of 16.5 s (average of 10 males) and a model with a lower period of 9 s. Both were broadcast simultaneously and with equal RMS amplitudes. Frequent transitions between the soft and loud song segments may be differently attractive for females. Finally, female phonotaxis was tested in a choice between two identical AM-song models, which alternated in the phase relationship between loud and soft segments. For this experiment, the frame rate of video recordings was increased from 12 to 20 frames per minute to improve the temporal resolution for the reconstruction of the female walking paths. This experiment should reveal whether females respond in a dynamically way to the alternating loud segments of AM-motifs in simultaneously singing males.

### Computer simulations of temporary bout and segment overlap

In male-male song interactions, males often tend to sing at the same time, so that song elements overlap with those of a competing male. To generate expected values for (1) accidental song bout overlap, and (2) for the accidental overlap of loud segments within two overlapping AM-motifs, two different simulations in the multi-agent simulation environment NetLogo 5.0.1. (Wilensky, U. 1999; https://ccl.northwestern.edu/netlogo/) were created. Simulated song interactions were based on parameters that had been observed during real acoustic interactions of males.

1. To discriminate events of intended temporal bout overlap from accidental ones, we simulated the latter situation to compare values with those of actual recorded male interactions. Bout overlap in simulated acoustic interactions is the consequence of randomly timing bouts with bout durations and total simulation time as observed in real acoustic interactions. The duration of simulated song bouts and pauses in between showed a Gaussian-like distribution and had average durations of 394.2 ± 61.4 s SD and 1080 ± 61.4 s SD, respectively. Simulation runs were stopped after 240 min (according to the same recording time in real interactions), and the frequency of overlapping bouts was evaluated after 100 simulation runs.
2. Second, we simulated the percentage of segment overlap during overlapping AM-motifs. We simulated 51 duets of NetLogo agents, whereby every single duet was based on parameters observed in acoustic interactions of two males. The duration of loud and soft song segments was simulated with a Gaussian-like variability, and the average segment duration was varied according to real observed values. The average degree of segment overlap was evaluated in two different simulation scenarios: either both agents started the interaction with loud segments or one agent started with a loud segment, the other with a soft segment. Each duet was simulated 20 times in both scenarios and means of all 102 simulated duets were averaged to achieve one significant value of random segment overlap.

### Statistical analysis

The dataset for multivariate statistical analysis was generated by averaging parameters that describe song bouts of individual males in three different situations. The within-male average of bout duration, the proportion of the AM-motif, the proportion of the trill, and the duration of loud and soft segments were used as input of a multivariate model that should be able to predict the situation in which songs were recorded. R studio (Version 1.0.44; RStudio, Inc.) was used for the development of a generalized linear regression model with log transformed song parameters as input. Model selection was made by comparing the AIC (Akaike’s Information Criterion) values of various different regression models that were fitted by calculating the maximum likelihood (Laplace Approximation) with the lme4 package of the R statistic software. The best GLMER model is shown in Eq. 1 and allows to predict the situation in which males produced their songs by combining four song parameters (AIC = 30.9; median of scaled residuals = 0.09). In this model, male ID acts as random term.

1 $$ \mathrm{Situation}\sim \mathrm{Bout}.\mathrm{duration}+\mathrm{Proportion}.\mathrm{AM}+\mathrm{Loud}.\mathrm{segment}+\mathrm{Soft}.\mathrm{segment}+\left(1\ |\ \mathrm{MaleID}\right) $$

In addition, principal component analysis was performed in SigmaPlot (Version 13.0, SPSS Inc.) to study the number of factors that are optimal for the explanation of data variance. Component loadings and the Scree test confirmed that the combination of at least four song parameters is optimal to explain data variance.

Because the same set of males was used in the isolation and acoustic interaction experiments, we developed a GLMER model using male ID as a random term to predict the social singing situation (see Eq. 2; AIC = 30.3; median of scaled residuals = 0.08).

2 $$ \mathrm{Situation}\sim \mathrm{Proportion}.\mathrm{AM}+\mathrm{Loud}.\mathrm{segment}+\left(1\ |\ \mathrm{MaleID}\right) $$

Since a different set of males was used to study songs in the presence of females, a GLM model (see Eq. 3) was developed to predict the singing situation on the basis of song parameters that were recorded either during isolation or in the presence of females (AIC = 17.3; median of residuals = 0.053).

3 $$ \mathrm{Situation}\sim \mathrm{Proportion}.\mathrm{AM}+\mathrm{Loud}.\mathrm{segment}+\mathrm{Soft}.\mathrm{segment} $$

To test for the significance of the female preference in a choice situation, a generalized linear mixed (GLM) model was developed in “R.”

$$ \mathrm{Binomial}\ \mathrm{GLM}\ \mathrm{model}:\mathrm{preferred}\_\mathrm{side}\sim \mathrm{stimulus}\_\mathrm{side}+\left(1|\mathrm{female}\_\mathrm{ID}\right) $$

This model allows testing the hypothesis that stimulus side influences female choice. Model fit was made by Laplace approximation assuming a binomial distribution of data. By treating female ID as a random intercept in this model, it was possible to handle the data replication that resulted from testing individual females several times in the same stimulus situation. Further statistical tests (*t* test, *z* test) were performed in SigmaPlot (Version 12.0, Systat Software Inc., Chicago, IL, USA). Before applying statistical tests, the Gaussian distribution of the data was tested by performing a Shapiro-Wilkinson test.

#### Data availability

The datasets analyzed during the current study are available from the corresponding author on reasonable request.

## Results

### Song characteristics

Song bouts of this species comprise two motifs, which differ mainly in the amplitude, but also in the fine structure of syllables. While the AM-motif consists of alternating loud and soft segments, the trill has a continuous high amplitude of 103.2 ± 1.7 dB SPL at a distance of 15 cm to the male, in contrast to 86.0 ± 3.9 dB SPL for soft segments. The same high amplitude was recorded for the loud segments of the AM-motif. Both, the loud song segments in the AM-motif and the trill consist of syllables that are twice as long as those in the soft segments (28.2 ± 1.55 ms vs. 14.3 ± 1.83 ms, *n* = 13; Fig. 1c). The spectrum in the song covers a wide frequency range, which includes both audio and ultrasonic frequencies up to 80 kHz. A frequency analysis has shown that both song motifs exhibit similar spectral compositions (Kostarakos and Römer 2015).

### Singing in different social contexts

**Fig. 3:**
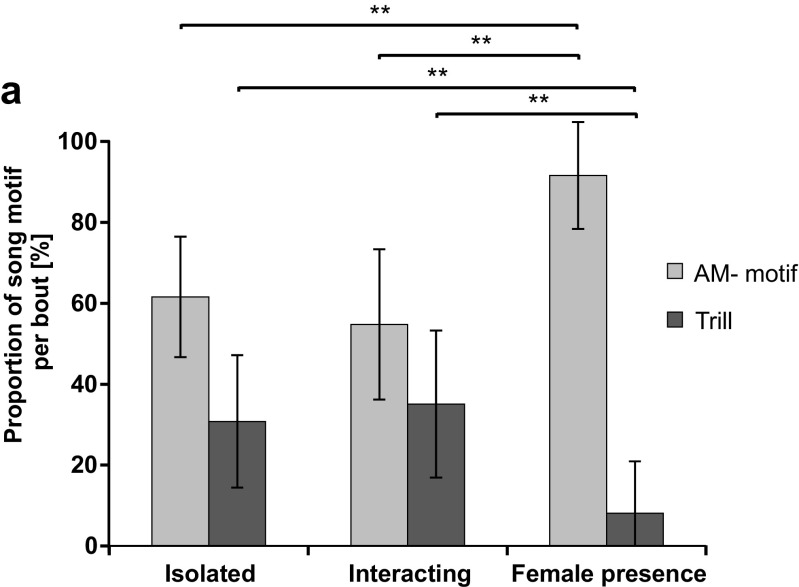
Temporal organization of song bouts; proportions of the two different motifs. **a** Average proportion of song motifs of solo singing males, pairs of two acoustically interacting males, and males singing in the presence of a female. Error bars indicate SD

Songs of males were recorded in three situations: in isolation, during acoustic interaction with another male, and in the presence of a female. Multivariate statistical analysis using Eq. 1 revealed that the proportion of the AM-motif and the duration of loud segments of AM-motifs have a significant effect on predicting the social context of singing in males (*p* < 0.05; *z* values = 2.35 and 2.23, respectively). In the presence of females, males significantly increased the proportion of the AM-motif (in relation to the total bout duration) compared to both other situations [(1) isolated males 57.3% ± 13.5, *n* = 14; (2) interacting males 54.8% ± 18.6, *n* = 10; (3) presence of a female 91.6% ± 13.2; *n* = 10; one-way ANOVA followed by a Tukey’s post hoc test: *p* < 0.001 between (3) and (1, 2); *p* = 0.920 between (1) and (2), Fig. 3)]. As a consequence, the proportion of trills relative to bout duration was reduced by males singing next to a female [(1) isolated males 30.6% ± 16.3, *n* = 14; (2) interacting males 35.1% ± 18.2, *n* = 10; (3) presence of a female 8.1% ± 12.7; *n* = 10; one-way ANOVA followed by a Tukey’s post hoc test: *p* < 0.01 between (3) and (1, 2); *p* = 0.772 between (1) and (2), Fig. 3)]. The GLM model shown in Eq. 3 confirmed this result and identified the proportion of the AM part as the only parameter that changed significantly when males were singing in the presence of females compared to solo singing (*p* < 0.05, *z* value = 2.06). The occurrence of song bouts longer than 1000 s was rare (about 10%) and almost identical in males singing in isolation and interactions with other males, but was never observed in the presence of females.

AM-motifs produced by the same males during acoustic interactions were significantly shorter compared to solo singing (interaction 196.6 ± 52.6 s, isolation 239.0 ± 82.4 s *p* < 0.05, paired *t* test), whereas the trill duration was almost identical in both situations (interaction 144.7 ± 94.8 s, isolation 143.4 ± 86.6 s *p* > 0.05, paired *t* test).

The average duration of soft segments was similar in all three situations ((1) isolation 7.3 ± 1.7 s vs. (2) interaction 7.4 ± 1.7 s, *p* = 0.784, paired *t* test, *n* = 10; (1) vs. (3) female presence 7.38 ± 1.25 s, *p* = 0.941, *t* test, *n* = 10; (2) vs. (3), *p* = 0.939, *t* test, *n* = 10). However, loud segments were significantly shorter during acoustic interactions compared to solo singing males ((1) 6.6 ± 1.3 s vs. (2) 5.4 ± 1.3 s, *p* < 0.001, paired *t* test, *n* = 10), but there was no difference between males singing in the presence of a female and the other two situations ((3) 5.73 ± 0.89 s vs. (1), *p* = 0.083, *t* test, *n* = 10; (2) vs. (3), *p* = 0.575, *t* test, *n* = 10). The GLMER model shown in Eq. 2 confirmed the significant change of the duration of loud segments when males were singing in isolation compared to song interaction (*p* < 0.05, *z* value = 1.97).

**Fig. 4:**
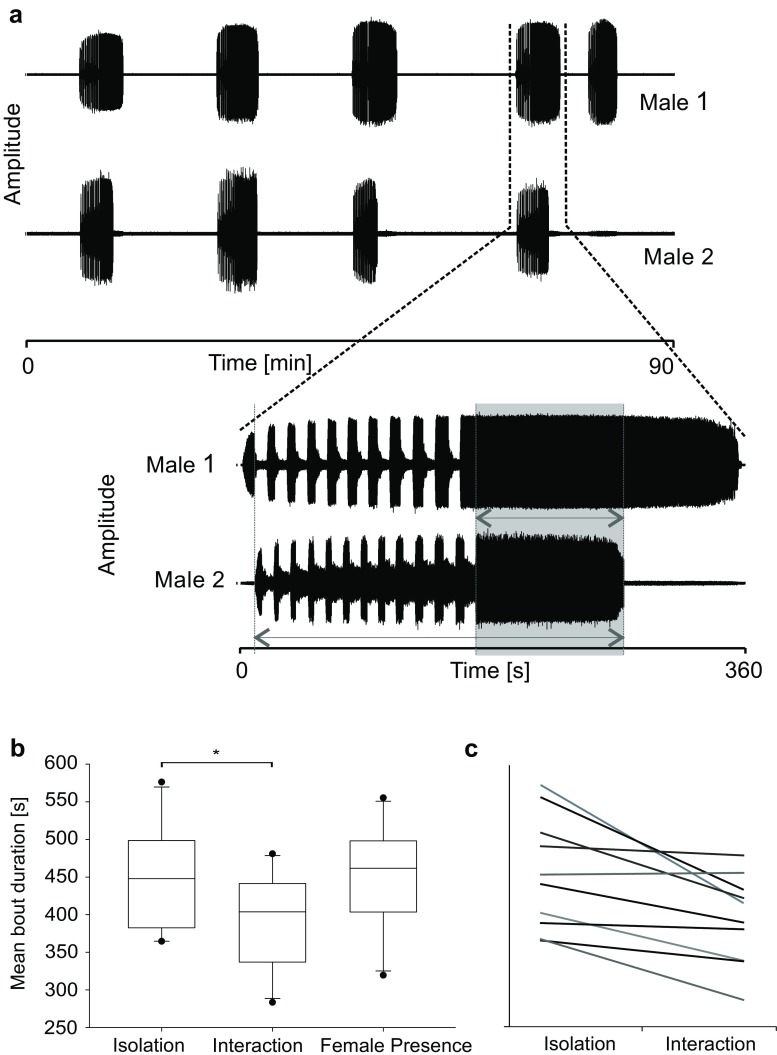
Acoustic interaction between two males singing within hearing range and comparison of bout duration. **a** Oscillogram of a 90-min recording of two males singing. Note the high degree of bout overlap. Loud segments of the AM-motif were not synchronized (magnification of one singing bout below); gray shaded area shows the time of trill overlap of both males. The longer arrow indicates the time taken as 100% reference for the calculation of trill overlap. **b** The average bout duration of the same males was significantly higher when singing in isolation than in interactions (*p* < 0.01, paired *t* test). When comparing all three groups with a one way ANOVA, no difference could be found. **c** Average bout duration of individual males in both situations

There was no difference in the mean song bout duration between all three situations ((1) isolation 447.8 ± 69.4 s vs. (2) interaction 394.2 ± 61.4 s vs. (3) female presence 450.3 ± 68.6 s, *p* = 0.123, One way ANOVA, *n* = 10). However, when considering the fact that in the first two situations, the sample of males was the same, statistical analysis using the paired *t* test revealed that the mean song bout duration of males was significantly higher when they were solo singing as compared to that during interactions (*p* < 0.01, paired *t* test, 93 solo bouts, 180 bouts during interactions, *n* = 10 males, Fig. 4b, c).

In the natural habitat of *Mecopoda* sp. males usually sing within earshot of other conspecific males, so that the probability of song overlap should be rather high. In our recordings of interacting males, bout overlap occurred with a probability of 58.3%, which is significantly higher than a theoretical overlap of 25.7% found in the simulation with independently singing virtual males (binomial test, *p* < 0.001). The average temporal overlap of song bouts amounts to 39.0% of the time period in which at least one male was singing. In the example shown in Fig. 4a, the temporal bout overlap was 58.4%, indicating that the males were highly motivated to sing simultaneously. The highly non-random nature of the bout overlap is also evident in the rather long and irregular periods of silence, that were maintained by both males between song bouts (isolated males 1516 ± 1322 s, *n* = 79; interacting males 1454 ± 1223 s, *n* = 162). The average amount of temporal overlap of loud segments within AM-motifs in pairwise interacting males was 16.5% of the total duration of the AM-motif. Assuming for a natural duration and variability of loud and soft song segments, NetLogo simulations revealed that 18% of loud song segments synchronized by chance (102 simulated duets). Thus, we could not confirm an intended overlap of loud song segments.

In acoustic interactions, trills overlapped on average 20.36 ± 18.8% of the time of song bout overlap (see also Fig. 4a). We also evaluated whether males changed the proportion of the trill motif during acoustic interactions when bouts of competitors either overlapped or not, but no differences were observed (mean proportion of the trill-motif relative to bout duration: 41.3% in overlapping bouts and 42.6% in non-overlapping bouts; *z* test; *p* > 0.05).

Table 1 summarizes all important statistical parameters of songs produced by males when singing in the three different social contexts. Values represent the mean ± SD of averaged parameters.

**Table 1:** Song statistics

Experiment	Bout duration [s]	Proportion of AM-part [%]	Proportion of trill [%]	Duration loud segments [s] (bouts/males)	Duration soft segments [s] (bouts/males)	Proportion of singing^a^ [%]
Solo singing	441.4 ± 89.2 (14 males)	57.3 ± 13.5 (14 males)	30.6 ± 16.3 (14 males)	6.6 ± 1.3 (144/10)^b^	7.3 ± 1.7 (144/10)	28.5 ± 13.4 (10 males)
Acoustic interactions	394.2 ± 61.4 (10 males)	54.8 ± 18.6 (10 males)	35.1 ± 18.2 (10 males)	5.4 ± 1.3 (102/10)	7.4 ± 1.7 (102/10)	24.3 ± 10.7 (10 males)
Female present	450.3 ± 68.5 (10 males)	91.6 ± 13.2 (10 males)	8.1 ± 12.7 (10 males)	5.7 ± 0.89 (17/10)	7.4 ± 1.25 (17/10)	n.a.

*n.a.* not available or not applicable

^a^Over 4 h of recording time

^**b**^144 bouts recorded from 10 males

### Female choice experiments

The preference of females for various song parameters was investigated in two-choice arena experiments (oscillograms of playback signals are shown in Fig. 2a). When given the choice between the continuous loud trill and the AM-motif, females approached the trill in 74% of trials (37 vs. 13 approaches; GLM model, *p* < 0.01, *n* = 50 trials by 13 females, Fig. 2c). However, after equalizing the RMS amplitude of both song models by decreasing the amplitude of the trill, the choice of speakers was random (49 vs. 51%, GLM model, *p* > 0.05, *n* = 47 trials by 13 females). In another set of trials, females were given a choice between two song models with a sequence of syllables that were typical for either loud or soft song segments of the AM-motif (see Fig. 1 for the syllable pattern). Females significantly preferred the syllable pattern that was typical for loud trills (syllable duration = 28 ms), although both song models were broadcast with equal RMS amplitude (75 vs. 25%, GLM model, *p* < 0.01, *n* = 28 by 8 females, Fig. 2c). Females also significantly preferred the high duty cycle song model of the AM-motif over the medium duty cycle model (GLM model, *p* < 0.05, *n* = 44 trials by 12 females). Nevertheless, the high duty cycle song model was equally attractive as compared to the low duty cycle song model (58 vs. 42%, GLM model, *p* > 0.05, *n* = 48 trials by 15 females), although both models were broadcast with the same maximum amplitude of loud segments. We also tested AM-song models with the same duty cycle of loud song segments, but with different durations of the period that included one loud and one soft segment (period 16.5 vs. 9 s). In this case, the majority of females preferred the stimulus with the shorter period (70 vs. 30%), which is significant according to the *z* test (*p* < 0.05 after Bonferroni correction), but is not significant in “R” when female ID was taken into account (GLM model, *p* = 0.111, *n* = 23 trials by 12 females).

**Fig. 5:**
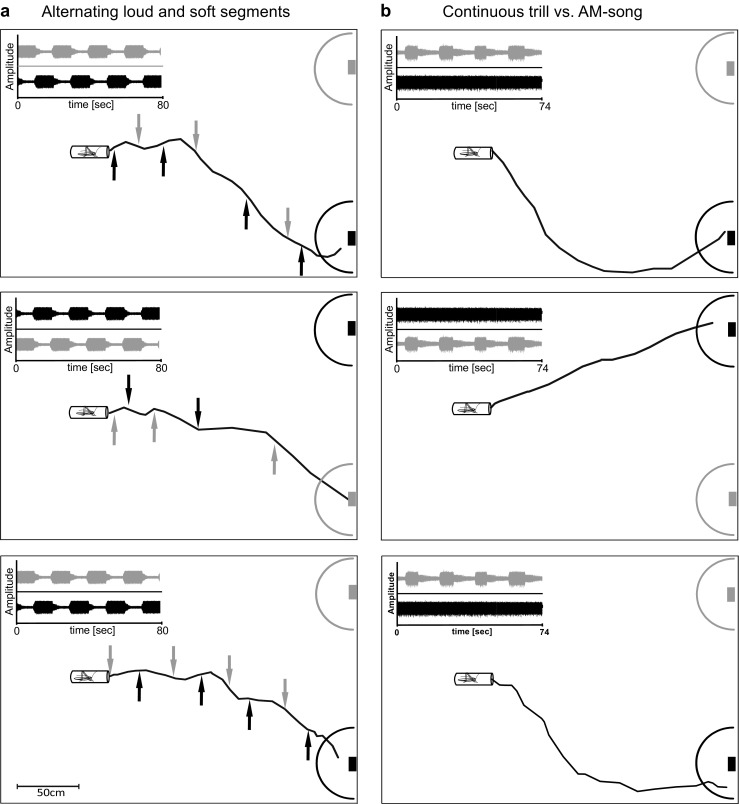
Walking paths of a representative female in two different choice situations. **a** When given the choice between two AM-motifs presented in perfect alternation, the female frequently changed her walking direction before heading toward a speaker (left panel). Arrows indicate the onset of the loud song segment in the corresponding speaker. **b** The same female selected the trill over the AM-motif presented at the same time from the other speaker (right panel). Note that in this choice situation, the female walking path did not show systematic changes in walking direction

Only 16.5% of the loud segments in the AM-motif overlapped in interacting males, which does not differ from random overlap. Thus, the loud segments of one male often alternated with the soft segment of the competitor. Therefore, we studied the phonotaxis of females in trials in which the same AM-motif was broadcast in alternation from two spatially separated speakers. Three examples of female walking paths are shown for this choice in Fig. 5 (for the sequence of song segments, see inset in panels of Fig. 5). The reconstruction of walking paths revealed a dynamic reorientation of females in this complex choice situation, particularly during the first third of the approach. Distant from the speakers at the beginning of the phonotactic trial, females changed their walking direction shortly after a speaker began emitting a loud segment, so that they appeared to be walking in a zig zag line toward the midpoint between the speakers. Closer to the speakers, however, females approached one speaker irrespective of which one was broadcasting the loud segment. For comparison, the phonotactic paths of the same female given the choice between the trill and the AM-motif are shown in Fig. 5b; the female always approached the speaker broadcasting the trill.

## Discussion

In this study, we tested the hypothesis that males modify their signaling behavior depending on the social environment (presence or absence of females and competing males) (Bretman et al. 2011; Rebar and Rodríguez 2016). Therefore, we recorded the acoustic signals produced by males in three different situations: when singing (1) solo, (2) together with another male, and (3) in the presence of a female.

Unlike many other katydids that produce a simple calling song consisting of the same, repeated elements, the song of the investigated *Mecopoda* species is remarkable due to its ear-deafening SPL and complex temporal structure, which consists of an AM-motif and a trill (Korsunovskaya 2008). The high SPL of the trill would indicate that the call functions to attract mates over long distances. It is likely that females would be able to detect the trill at distances of 50 m or more in their natural habitat, even when considering that attenuation in excess to geometric spreading may occur. This is likely because the low-frequency components of the call would suffer less attenuation as compared to the high frequency and ultrasound components (Römer and Lewald 1992). Males that do not receive sensory feedback indicative of the presence of a nearby female or competing male would be expected to produce songs that have far-reaching properties in order to attract as many females as possible. When males detect the presence of a female, they modify the song by increasing the proportion of the AM-motif to more than 90% (Fig. 3). Consistent with this strong increase in the AM-motif at the expense of the trill is the finding that extremely long song bouts (> 1000 s) consisting only of trills never occurred in the presence of females. The hypothesis that the AM-motif is directed to a female audience is further supported by the fact that the AM-motif, but not the trill, is accompanied by the production of distinct vibratory signals that have a strict temporal relationship with the loud and soft segments in the AM-motif (Erregger 2014). Yet, since the loud segments of the AM-motif are as intense as the trill, the AM-song cannot be considered to be part of a less conspicuous, private communication, as in the case of the “love song” during Drosophila courtship or the wagging dance of the honey bee, both using near-field sound for communication with a detection range of only a few millimeters (Tauber and Eberl 2003; Michelsen et al. 1987). Yet, the transmission of the vibratory signals during the production of the AM-motif in *Mecopoda* is limited to the substrate shared by the sender and receiver. Thus, the vibratory component has a rather limited detection range compared to the air-borne AM-motif. Its function can therefore be seen in the context of courtship, rather than long-range attraction.

We expected males that detected another singing male to modify their song to either (1) increase their own attractiveness to females and/or (2) decrease the detectability/attractiveness of competing signals. With respect to the first option, our female choice experiments revealed how males should have modified their song to become more attractive to females. We observed a general preference for loud trills and AM-motifs with a high duty cycle of loud segments. This is consistent with the findings in other acoustic insects and anurans where a preference based on small differences in signal rate (Trobe et al. 2011) and signal amplitude in the order of 2–3 dB was observed in choice situations (Gerhardt and Huber 2002). Males with higher calling amplitudes occupy a larger active space (the area over which males are able to attract females) and, thus, will be able to attract a higher number of females, given the passive attraction of females to more conspicuous signals (Parker 1983; see also the frequent reorientation of females to the louder of two competing signals in Fig. 5).

Besides passive mate choice, females may prefer the high amplitude trill due to the higher energetic demand that is associated with the production of this song motif. Thus females selecting calling songs that are energetically demanding to produce (trill or high duty cycle AM-motifs) may select males of higher fitness. Results of a metabolic study conducted with males that sing in a respirometer chamber demonstrate that the CO_2_-production rate is increased during the trill 12 times above the resting rate (Erregger et al. 2017). Since females also show a preference for a syllable pattern that typically consists of loud segments of the AM-part and trills, females seem to prefer song elements that are associated with higher energetic costs, which potentially provide them with information about the quality of males (Zahavi 1977). Energetic costs of calling songs are mostly determined by the sound pressure level, repetition rate, and call duration (Prestwich 1994). In *Neoconocephalus robustus* and *Euconocephalus nasutus*, high duty cycle calling songs are associated with an oxygen uptake rate that is comparable to the energy consumption of wing muscles during flight. In order to produce calling songs with extraordinary high pulse rates, *Neoconocephalus robustus*, *Euconocephalus nasutus* (Stevens and Josephson 1977), and *Hexacentrus unicolor* (Heller 1986) males warm up their thorax muscles prior to producing sound.

Surprisingly, however, in our study, males that interacted with another male neither increased the duration nor the proportion of song elements that are particularly attractive to females (trill and high amplitude part of AM-motif). Nevertheless, interacting males did not simply ignore other singing males, since the proportion of bout overlap observed was much higher than that one would expect during random signaling. Interacting males showed a high degree of bout overlap and reduced the duration of loud segments of the AM-motif while the duration of soft segments was comparable to that observed during solo singing. This behavior reduced the duration of the signal period of loud and soft segments and increased a male’s attractiveness in a choice situation (Fig. 2c, f). In most male-male interactions, we observed that males tended to sing at the same time. However, a correlation of bout duration, which would indicate the presence of an ongoing competition between the males in an attempt to jam each other’s signals, as reported in various other species (Sheridan et al. 1993, Greenfield 1994a, Tobias and Seddon 2009, Rebar and Rodríguez 2016), was only found in one male pair. The most parsimonious explanation for the observed bout overlap singing is that the male would suffer a disadvantage if he did not start singing when a competitor did. Proof for this explanation is provided in the female walking path toward two signals alternating in their loud segments (Fig. 5). A male that did not compete acoustically during the bout of a competitor would risk the female approaching the other male, since no other signal would be available for a choice. Thus, even though a male does not know whether females are present within the active range of his signal, he must compete with other males by bout overlap singing.

The fact that interacting males do not intentionally overlap the loud segments of the AM-motif (supported by the Netlogo simulation) does not suggest any cooperative effect which increases the sound output of a group of synchronously singing males compared to solo singers (Buck and Buck 1966, 1978; Greenfield 1994b; Hartbauer et al. 2014). Other group advantages may arise in the context of selection pressures by predators or parasitoids (Lehmann and Heller 1998, Zuk and Kolluru 1998), and future field studies must be conducted to find out what motivates males of this trilling species to spend a significant amount of their time signaling together in a chorus.

## Abbreviations

RMS: Root mean square
AM-motif: Amplitude-modulated motif
DC: Duty cycle
SPL: Sound pressure level
